# A Simple and Robust Fabrication Process for SU-8 In-Plane MEMS Structures

**DOI:** 10.3390/mi11030317

**Published:** 2020-03-18

**Authors:** Chang Ge, Edmond Cretu

**Affiliations:** Department of Electrical and Computer Engineering, University of British Columbia; 3063-2332 Main Mall, Vancouver, BC V6T 1Z4, Canada; edmondc@ece.ubc.ca

**Keywords:** polymeric MEMS, SU-8 micromachining, polymer substrate, maskless lithography, laser micromachining

## Abstract

In this paper, a simple fabrication process for SU-8 in-plane micro electro-mechanical systems (MEMS) structures, called “border-bulk micromachining”, is introduced. It aims to enhance the potential of SU-8 MEMS structures for applications such as low-cost/disposable microsystems and wearable MEMS. The fabrication process is robust and uses only four processing steps to fabricate SU-8 in-plane MEMS structures, simplifying the fabrication flow in comparison with other reported attempts. The whole fabrication process has been implemented on copper-polyimide composites. A new processing method enables the direct, laser-based micromachining of polyimide in a practical way, bringing in extra processing safety and simplicity. After forming the polymeric in-plane MEMS structures through SU-8 lithography, a copper wet etching masked by the SU-8 structure layers is carried out. After the wet etching, fabricated in-plane MEMS structures are suspended within an open window on the substrate, similar to the final status of in-plane MEMS devices made from industrial silicon micromachining methods (such as SOIMUMPS). The last step of the fabrication flow is a magnetron sputtering of aluminum. The border-bulk micromachining process has been experimentally evaluated through the fabrication and the characterization of simple in-plane electrically actuated MEMS test structures. The characterization results of these simple test structures have verified the following process qualities: controllability, reproducibility, predictability and general robustness.

## 1. Introduction

Micro electro-mechanical systems (MEMS) consist of sensors and actuators transferring information and energy between the electrical and mechanical domains. Capacitive coupling is one of the major coupling principles for both sensing and actuation of MEMS devices that serve as vital front-end devices for many modern microsystems. Based on their mechanical degree-of-freedom with reference to the substrate plane, MEMS devices can be categorized into out-of-plane and in-plane devices. 

Recently, polymeric MEMS devices have drawn attention from the academic community and industry for their potential in emerging applications such as low-cost/disposable electronics, wearable systems, and Internet-of-things [1]. As an example of this perspective, high performance polymeric capacitive micromachined ultrasound transducer arrays (CMUT) have been fabricated and validated for their use in ultrasound imaging [2]. The performance of the polymeric CMUT has been comparable with classic ultrasound transducers, but the fabrication complexity and cost has been significantly lower. 

**Figure 1:**
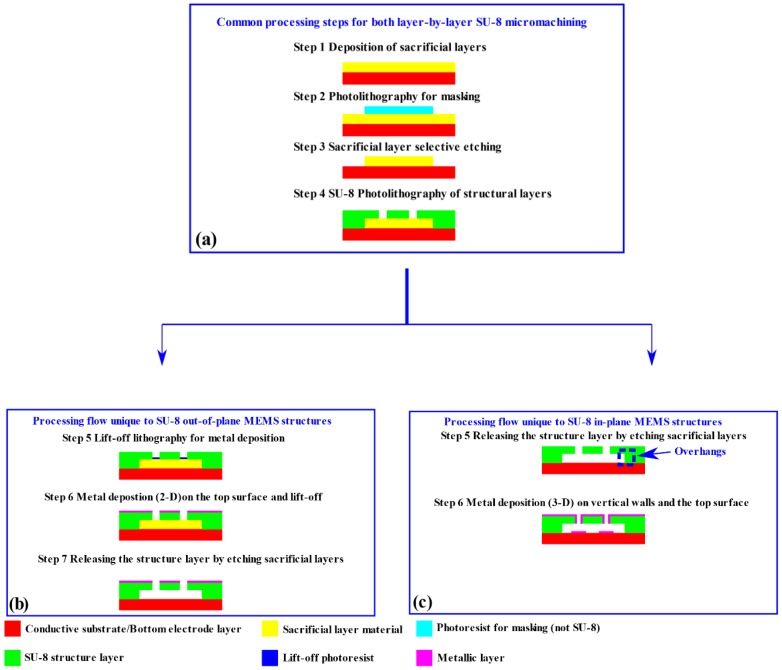
The generic schematics of layer-by-layer SU-8 micromachining. (**a**) Common steps; (**b**) Steps for out-of-plane SU-8 MEMS structures; (**c**) Steps for in-plane SU-8 MEMS structures.

The fabrication cost, speed and process simplicity are vital to polymeric MEMS structures and their applications. This explains why SU-8 series negative photoresist has been advantageous and popular as structural layers for polymeric MEMS devices. Such processes require only a single lithography process to form microstructures, with its simplicity leading to a low fabrication cost, while SU-8 has better mechanical properties and chemical stability, in comparison with other photoresists [3,4]. For the fabrication of both out-of-plane and in-plane SU-8 electrostatic MEMS structures, the most commonly used fabrication flow is layer-by-layer surface micromachining [5,6,7,8,9,10,11,12]. The generic schematic of a layer-by-layer SU-8 micromachining process is illustrated in Figure 1. 

As shown in Figure 1, for both the in-plane and out-of-plane SU-8 MEMS structures, the first four steps of the layer-by-layer surface micromachinings can follow the same route. For these steps, the process simplicity and flexibility are determined by the selection of the sacrificial layers. The processing flow depicted in Figure 1 can be used for non-photosensitive polymeric sacrificial layers, metallic sacrificial layers or silicic ones [2,13,14,15,16,17]. Other photoresist types can be used as sacrificial layers [6,8,18,19]. The corresponding selective patterning only requires a single lithography step, reducing the two steps from the common processing flow in Figure 1a.

Figure 1 also illustrates the differences between the surface micromachining processes for out-of-plane and in-plane SU-8 MEMS structures. The main difference lies in the sequence of the metallization and the release step. For out-of-plane devices [5,6,7,8,9,10], they only need a conductive layer on the top surface of the SU-8 structures as the top electrode, while the conductive substrate (or a bottom electrode patterning) serves as the second electrode for the coupling. Two-dimensional deposition methods of metals work well in this case. Thus, as shown in Figure 1b, selective metallization through methods such as lift-off are carried out first, to define the top electrodes. Then, the out-of-plane electrostatic SU-8 MEMS structures are released through the etching of sacrificial layers. 

For in-plane devices [11,12], due to the requirement to cover the polymeric vertical walls with a metal film (in order to achieve in-plane electrical actuation and sensing), only three-dimensional deposition methods, such as tilted E-beam evaporation or sputtering, are suitable. In order to avoid electrical short-circuits between the fixed parts of the device and the substrate, due to the three-dimensional coatings, as shown in Figure 1c, the release step has to define overhangs at the edges of the anchored electrodes (the parts circled in the blue frame). In such a case, the 3D metallization methods cannot fully cover the bottom surface of the overhangs, and electrical insulation is guaranteed. 

The classic layer-by-layer surface micromachining processes depicted in Figure 1 for out-of-plane and in-plane SU-8 MEMS structures have their own challenges. When the SU-8 surface micromachining in Figure 1 is used to fabricate out-of-plane polymeric electrostatic MEMS structures, the use of sacrificial layers often generates an increased process complexity. To address this problem, the authors have previously developed and experimentally validated fabrication flows for out-of-plane SU-8 MEMS structures, based on grayscale lithography techniques, on both rigid substrates and flexible ones [20,21,22]. The developed methods do not use any sacrificial layers during the fabrication, removing four steps from traditional SU-8 surface micromachining for out-of-plane MEMS structures. 

For SU-8 surface micromachining of in-plane polymeric MEMS structures [11,12], one of the main issues when very thin sacrificial layers are used is the difficulty of the release process. Without an accurate control of the technological step, stiction to the substrate can strongly affect the performance of the fabricated devices. In comparison, silicic in-plane MEMS structures fabricated using bulk micromachining do not have such problems—the substrate underneath the movable parts is completely removed, suspending the structures within the open windows on the substrate. This fabrication strategy has been common practice for standardized industrial fabrication of silicic in-plane MEMS structures, like in the SOIMUMPs technology [23]. In order to enhance the application potential of polymeric in-plane electrostatic MEMS structures based on SU-8 photoresist, a simple fabrication flow, which can suspend the movable components while removing the substrate below, becomes a desirable target.

**Figure 2:**
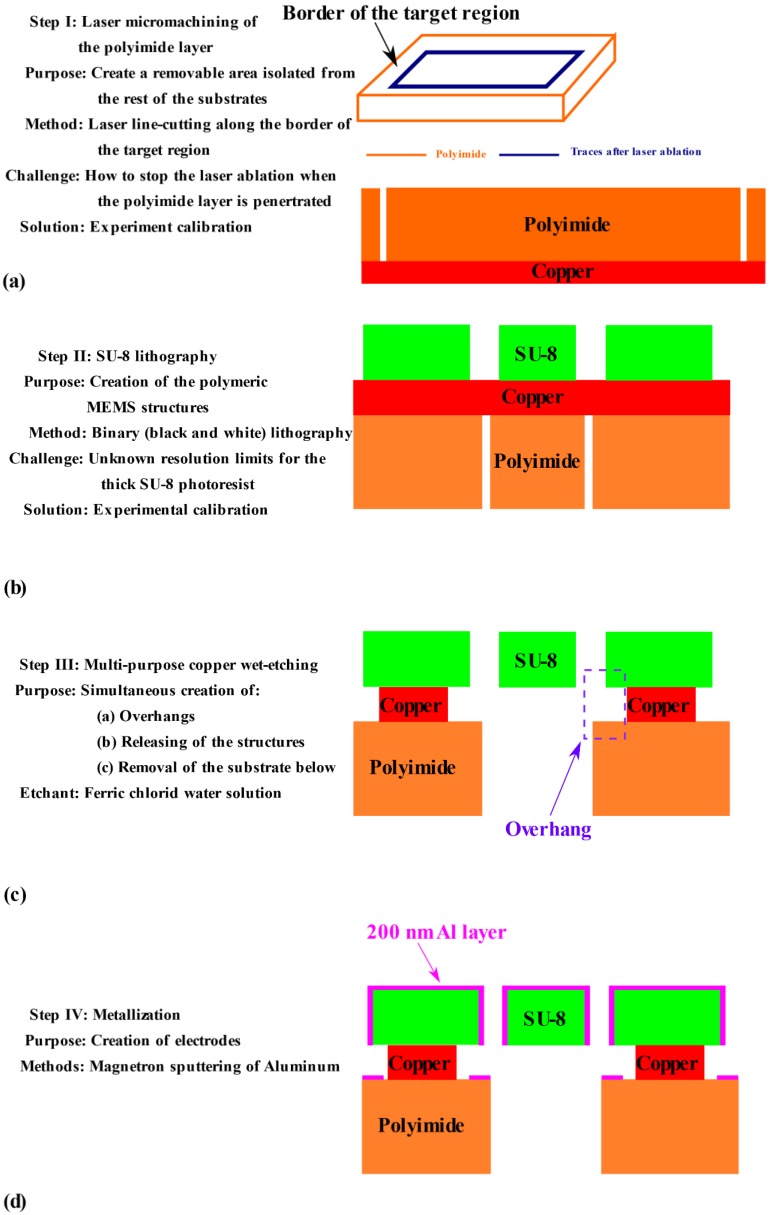
Illustration of the border-bulk micromachining process for SU-8 in-plane MEMS structures. (**a**) Direct micromachining of the polyimide layer; (**b**) SU-8 lithography on the surface of the copper layer; (**c**) Multi-purpose wet etching; (**d**) Metallization.

Consequently, the authors have developed a four-step micromachining flow for SU-8 in-plane MEMS structures, named “**border-bulk micromachining**”, as shown in Figure 2. Aimed at applications such as low-cost/disposable electronics and wearable MEMS, the border-bulk microfabrication process uses Pyrulax™ copper-polyimide composites (Dupont, Wilmington, DE, USA) as substrate materials. The polyimide layer has been patterned using a direct, rapid micromachining method, safer and simpler than the traditional methods [24]. The SU-8 lithography has been directly conducted on the copper surface of the composites, without any pre-processing of the copper layers. Then, a copper wet etching would simultaneously achieve the release of movable MEMS structures, the formation of overhangs, and the removal of the substrate underneath the in-plane movable structures. The released structures are suspended in an open window on the substrates. For metallization, an aluminum thin film is deposited onto the structures by tilted magnetron sputtering. 

In this paper, the newly designed processing flow has been experimentally validated through the fabrication of simple in-plane capacitive MEMS structures used as test devices. Through the characterization of the fabricated simple MEMS structures, the processing qualities of the newly developed four-step method has been evaluated. In the experimental case study, the technology is validated to have a good controllability, reproducibility, predictability, and robustness. In the rest of the paper, Section 2 introduces the generic idea of the border-bulk microfabrication. Section 3 presents the experimental setup used for the technology validation. The characterization results and their interpretation are provided in Section 4, while Section 5 draws the final conclusions. 

## 2. Design of the Fabrication Flow

The four-step “border-bulk” microfabrication flow is illustrated in Figure 2.

As shown in Figure 2a, instead of removing the entire targeted substrate region under the active (movable) structures, the newly developed method firstly isolates the region from the rest of the substrate, by etching only the border of the polyimide active area. This will make the polyimide-copper region removable in a subsequent step. After the polyimide border etching, the copper layer becomes exposed. During the copper etching as the third step of the whole flow, the polyimide within the targeted area will be disconnected from the rest of the substrate. The advantage of this processing method is the minimization of the impact of the etching step on the copper surface, avoiding the technology-induced degradation of the SU-8 lithography in the next step. 

For the polyimide border removal, laser micromachining becomes a better choice than other traditional methods, due to the associated process safety, simplicity, and acceptable processing duration. In comparison with the traditional patterning method of polyimide [24], laser micromachining, as a direct processing method, does not require the layer-by-layer surface micromachining of masking materials on polyimide surface; nor does it use any hazardous chemicals, such as potassium hydroxide. In one of the authors’ previous work, laser-based area milling has been used to define polyimide circular membranes [25], proving the feasibility of the process flow shown in Figure 2a. 

One common challenge concerning the efficiency of laser-based micro-milling for microfabrication is the required processing time. Laser micromachining is a serial process, where the laser beam moves along the desired path, removing the target material by thermal ablation. In area micromachining processes, such as the milling previously done by the authors [25], in order to remove the material within the entire targeted area, cutting paths are spaced from each other with the radius of the laser dot, filling the whole area with either zig-zag or concentric line patterns. Consequently, it takes hours to finish the total area removal process. However, when laser micromachining is used to implement the present alternative (border-etching) approach, the issues related to lengthy processing time are alleviated. In the newly proposed processing method, only the border of the region-to-removal need to be etched by the laser, significantly reducing the total length of the required cutting path, and resulting in a tremendous reduction of the processing time. The only extra step is a pre-calibration process, so that the laser cutting affects only the polyimide layer and not the copper one.

As shown in Figure 2b, the SU-8 lithography for in-plane MEMS structures is carried out without any pre-treatment of the copper surface. For the lithography process in Figure 2b, the only extra work to complete is the calibration of the SU-8 lithography on the copper surface, in order to determine the resolution limits and optimum exposure dose. The limits control the minimum obtainable distance between the vertical walls of SU-8 in-plane MEMS structures, which is a critical factor in obtaining larger coupling capacitance for the sensing and actuation of MEMS structures. 

The copper etching shown in Figure 2c implements three processing goals. By simply immersing the samples into a ferric chloride water solution, the creation of overhangs on the anchored regions (circled in purple in Figure 2c), the releasing of the in-plane structures, and the removal of the polyimide substrate below, are simultaneously achieved, leaving the structures suspended in an open window on the substrate and electrically isolated. In comparison, in traditional fabrication flows for SU-8 in-plane MEMS structures, multiple fabrication steps are necessary for achieving these goals [11,12], with an extra lithography process to create the overhangs. In addition, existing fabrication flows mainly mimic silicon-based surface micromachining, leaving the substrate under the in-plane structures untouched. 

For the metallization based on magnetron sputtering shown in Figure 2d, the only potential risk is to not achieve complete coverage of the vertical walls, due to the large aspect ratio of the structures. In an existing work [12], the solution to this issue has been tilting the sample in accordance with the aspect ratio of the parallel plates. The tilting angle can be computed by:

(1) $$\theta =\arctan(\frac{g}{H})$$

In Equation (1), *g* is the actual gap obtained during the SU-8 lithography, while *H* is the thickness of the vertical walls.

## 3. Set Up for Experimental Validation

To validate the novel processing flow in Section 2, fabrication and characterization of test in-plane SU-8 capacitive MEMS structures has been carried out. The Pyrulax™ composite ordered from Dupont consists of a 45 µm thick polyimide and 25 µm thick copper layers. SU-8 in-plane MEMS structures have been fabricated using a 75-um thick SU-8 2050 layer. 

As mentioned during the discussion of the first (Figure 2a) and second step (Figure 2b), when a specific type of Pyrulax™ composite and SU-8 layer have been selected for the fabrication, one-time experimental calibrations for laser micromachining on polyimide and SU-8 lithography on copper surface are necessary, in order to determine the corresponding design rules. 

### 3.1. Calibration of Laser Micromachining and SU-8 Lithography

**Figure 3:**
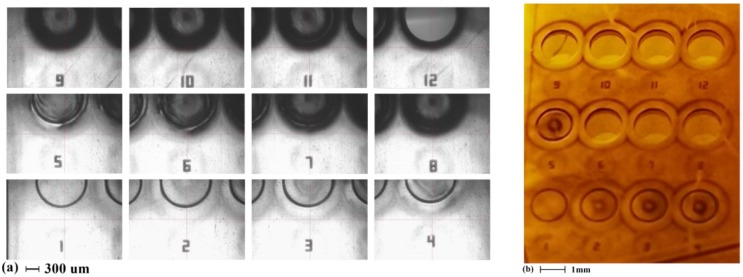
Calibration result for the laser micromachining process of polyimide. (**a**) After the laser micromachining; (**b**) After the copper etching.

In this work, the laser micromachining of the polyimide was performed using an Oxford^®^ laser micromachining system (wavelength: 355 nm, Oxford Lasers, Shirley, MA, USA). During the calibration process, the relative output power was fixed at 85%, while the moving speed of the laser dot was set at 1 mm/s. A set of 500 µm (diameter) circles were used as calibration test structures, each with a different number of laser cut cycles, so that the optimum number of cycles could be identified (optimum in the sense that the polyimide layer is completely cut through without affecting the Cu layer). After the laser micromachining, the processed samples are immersed in the copper etchant. The performance evaluation criterion is the visual detection of the disconnection of the circular plate from the rest of the polyimide film. The calibration result is shown in Figure 3. 

In Figure 3, the number of laser cut passes associated with each test structure was inscribed on the film. Shown in Figure 3a, for a Pyrulax™ composite with a 45 µm polyimide layer, both the copper layer and the polyimide layer would be penetrated when the laser cut was repeated 12 times. When the cut was repeated 6 times, the profile of the circle became significantly blackened. As shown in Figure 3b, for cuttings repeated more than 6 times, the corresponding circular plates were disconnected from the substrate. Thus, these cuttings have penetrated the polyimide layer, reaching the copper layer, while the blackened profile in Figure 3a can be used as an indication for the penetration of the polyimide layers. 

The SU-8 lithography process in this work was conducted using an Advanced Micro Patterning^®^ SF-100 maskless lithography system (equipment resolution: 0.6 µm, labeled output intensity @ 365 nm: 10 mW/cm^2^, Advanced Micro Patterning LLC, Delray Beach, FL, USA). The parameters for soft baking, post-exposure baking and the developing process of the 75 µm SU-8 2050 are summarized in Table 1. 

**Figure 4:**
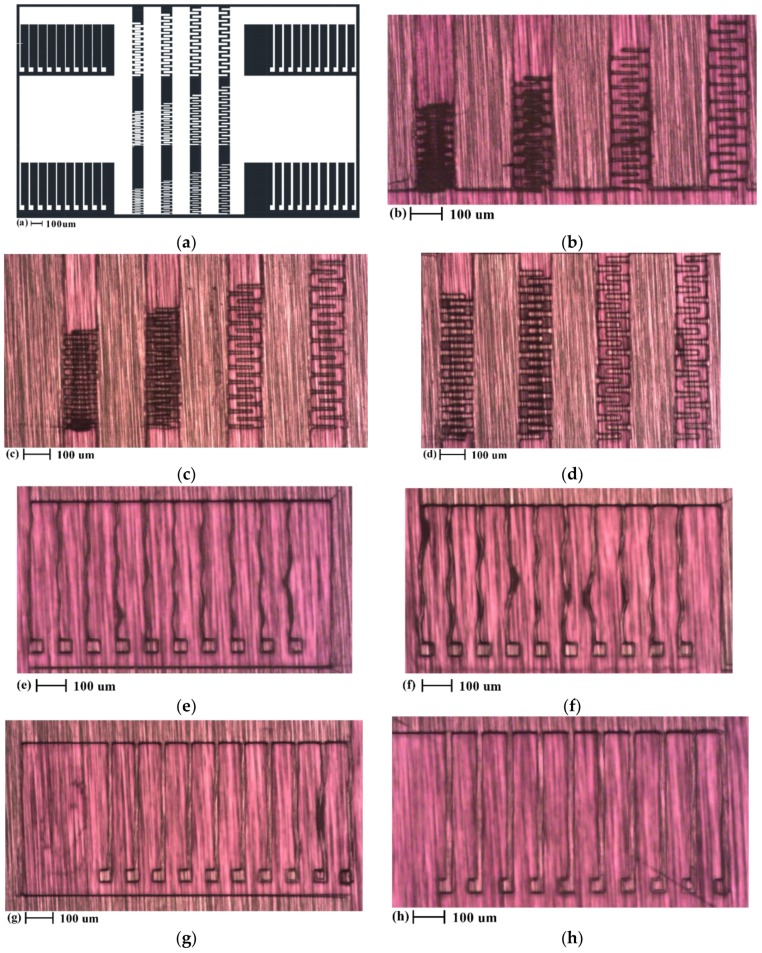
Mask design and the result of the SU-8 lithography calibration process. (**a**) The mask design for the calibration; (**b**) to (**d**): Representative lithography result for interdigitated plates with different plate width. (**b**): 5 µm, (**c**): 10 µm, (**d**): 20 µm. (**e**) to (**f**): Representative lithography result for beams with different width, (**e**): 5 µm, (**f**): 10 µm, (**g**): 15 µm, (**h**): 20 µm.

The calibrated parameter is the exposure duration, ranging from 5 seconds to 10 seconds, with 0.2-second increments. As a commonly practiced method for parameter-controlled microfabrication, the processing parameters in Table 1, once calibrated, are also used in the actual fabrication processes. The calibration mask design and the calibration result are shown in Figure 4. 

The calibration mask in Figure 4a was designed using AutoCAD 2017 (Autodesk, San Rafael, CA, USA). The white pixels were exposed to UV radiation. For the long beams in Figure 4a, their length was kept constant at 400 µm, while their width varied from 5 µm to 20 µm. The calibration target was to get a minimum width for non-distorted beam structures, primitive elements that can serve as springs in the proof-of-concept design. For the comb drives in Figure 4a, their length was kept constant at 125 µm while their width varied from 5 µm to 20 µm. For each width value, four gap distance values, 10 µm, 15 µm, 20 µm, and 25 µm, were used to space the fingers, and to determine the minimum achievable gap. 

It was found that exposure durations between 7 s to 8 s could provide the most balanced results between structural quality (no deformation) and the lithography resolution. The representative result shown in Figure 4b–d corresponds to this range of exposure duration. As shown in Figure 4b–d, the first designed gap distance that led to a robust separation between the comb fingers was 20 µm; nevertheless, there is a gap narrowing effect of 5 µm from each finger side, resulting in a nominal gap distance of 10 µm. For fabrication technology of in-plane capacitive MEMS structures, the minimum achievable gaps between parallel plates is an important criterion for the process capability. The 10 µm minimum gap obtained here compares favorably with minimum achievable gap distances obtained by other polymer-based technologies for in-plane MEMS devices [11,12,26]. As for the narrowest beam for the springs, as shown in Figure 4h, a width value of 20 µm has been found to be the most robust design. 

### 3.2. Design of the Simple in-plane SU-8 MEMS Structures to Validate the New Fabrication Flow

**Figure 5:**
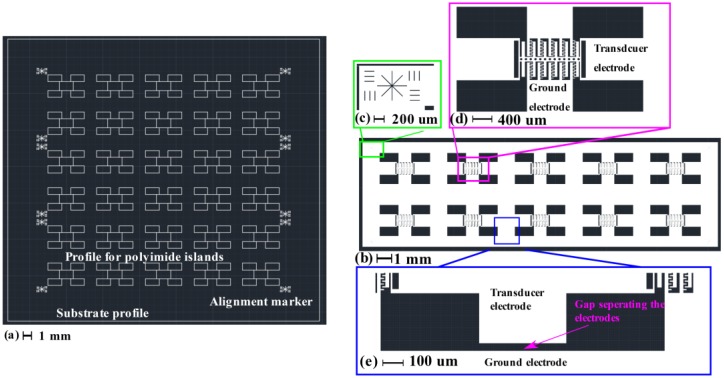
Mask files used to fabricate the in-plane MEMS test structures. (**a**) Mask for laser micromachining of the polyimide layers; (**b**) Lithography mask for the test structures; (**c**) Zoom-in view of the alignment markers; (**d**) Zoom-in view of MEMS structures; (**e**) Zoom-in view of the gap separating the transducer electrodes from the ground electrodes.

Using the calibration results shown in Figure 4 as design rules, a simple in-plane SU-8 capacitive MEMS test structure was designed for fabrication. The structural information is summarized in Table 2, while the mask design for the structures is shown in Figure 5.

**Figure 6:**
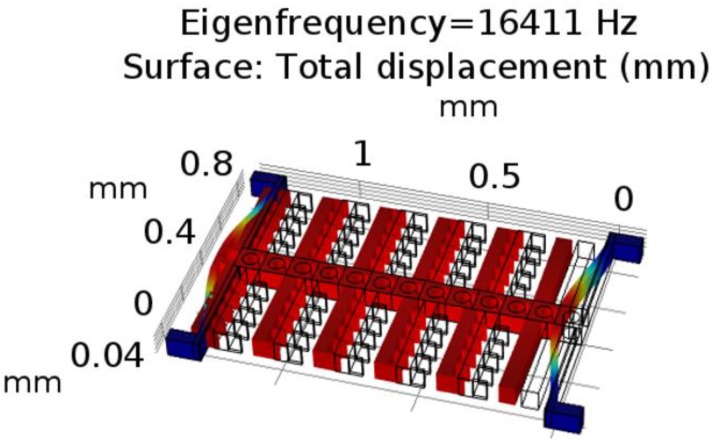
COMSOL simulation results of the fundamental resonant mode for the simple in-plane test structures.

For the laser micromachining mask in Figure 5a, the white lines correspond to the designed path for the laser cut. As shown in Figure 5a, the size of the Pyrulax™ composite used as substrate was 30 mm by 33.4 mm. On the surface of the polyimide layer, 30 ‘islands’ isolated from the rest of the substrate were created by laser cutting along their borders, corresponding to 30 in-plane SU-8 MEMS structures on the copper surface. For these ‘islands’, the laser path only needs to etch the polyimide layer. At the corners of the ‘islands’, markers were designed to help the alignment during the lithography on the copper surface. For these markers, the laser pass must penetrate both the polyimide and the copper layers, to make the alignment makers visible on the copper surface. The alignment marker design in Figure 5c corresponds to the laser-micromachined markers in Figure 5a. For one of the proof-of-concept structures, Figure 5d shows the implementation of the area-varying comb drives ensuring the electro-mechanical coupling. Release holes were spaced by 100 µm from each other on the movable parts. To avoid electrical short-circuits between different electrodes, as shown in Figure 5e, a 50 µm gap was designed between the transducer electrodes and the ground electrodes. For the proof-of-concept structures, the simulation result of their mechanical fundamental resonant frequency, to be used in the evaluation of process predictability, is shown in Figure 6.

### 3.3. Fabrication of the Simple in-plane Test Structures 

Using the masks in Figure 5, two batch fabrication iterations were processed, with a total of 60 in-plane capacitive MEMS test structures fabricated. 

For the laser micromachining using the mask shown in Figure 5a, the relative output power was kept at 85%, and the laser speed at 1 mm/s. The laser cut was repeated 7 times to create polyimide ‘islands’ isolated from the rest of the substrate, while for the drilling of alignment markers, the laser cut was repeated 14 times. 

For the SU-8 lithography using the mask in Figure 5b, an exposure time of 7.5 s (75 mJ/cm^2^ according to labelled intensity of 10 mW/cm^2^) was selected, based on the calibration result shown in Figure 4. The other process parameters were the same as the ones in Table 1. In order to carry out the spin-coating process for 75 µm SU-8 2050, the polyimide sheet was fixed on a 4-inch silicon wafer by Kapton tapes. 

The releasing of the structures was done by immersing the samples in copper etchant purchased from MG Chemicals (labeled etch rate: 4.4 to 5 µm/min) for 5 h. 

200 nm Aluminum was deposited afterwards onto the structures by tilted magnetron sputtering using an AJA^®^ thin film deposition system (AJA International Inc., North Scituate, MA, USA). After considering the 5 µm resolution degeneration, the 25 µm design was expected to result in a 15 µm actual gap. Thus, for the 75 µm vertical walls, the tilting angle was computed to be 11.3°.

### 3.4. Characterization Techniques

First, the fabricated structures were optically inspected under a microscope. The primary purpose was to validate the formation of the overhangs, evaluating the actual **robustness** and **controllability** of the fabrication process. 

Then, the electro-mechanical coupling behaviors of the fabricated simple in-plane structures were characterized to evaluate the qualities of the newly developed fabrication technology. First, the mechanical resonance of individual test structures under electrostatic actuation was measured. For each of the two batches, 15 out of the 30 test structures were randomly selected for the measurement of mechanical resonance, using the planar motion analyzer module of a Polytec^®^ MSA-500 system. The actuation signal consists of 5 V DC bias with an additional 3 V peak-to-peak AC chirp wave. The signal was amplified by 20 times using an A Tegam^®^ 2350 amplifier (TEGAM, Geneva, OH, USA), before being used as electrical actuation voltage for the comb drives. The electrical actuation validates the existence of metal coating on the vertical wall. The standard deviation of the measured frequency was used to evaluate the **reproducibility** of the developed fabrication process, while the average resonant frequency reflected the process **predictability**. 

For the second characterization test of the electro-mechanical coupling behaviors, the overall electrical impedance value of all 60 fabricated test structures connected in parallel during the electrostatic actuation was measured. The impedance measurement has two purposes: firstly, to validate the existence of the mechanical-to-electrical coupling by inspecting the back-reflection of the mechanical resonance on the electrical impedance curve (for a frequency band centered around the mechanical resonant frequency); secondly, to evaluate the degree of coverage of the metal thin film on the vertical walls, by comparing the measured the electrical impedance magnitude with the estimated one. For the measurement fulfilling the second purpose, a frequency band away from the mechanical resonant frequency was selected, to minimize the effect of electro-mechanical coupling. A good coverage should result in a measured capacitance value close to the estimated one for 100% vertical wall metal coverage. An Agilent^®^ A4294A impedance analyzer (Agilent, Santa Clara, CA, USA) was used for the impedance measurement, with the input excitation signal consisting of a 40 V DC bias and a 2 V peak-to-peak AC chirp wave. 

Through the measurement of both mechanical resonance and electrical impedance under the electrostatic actuation, the complete bidirectional electro-mechanical coupling was checked. The measurement of the mechanical in-plane motion under electrical actuation validated the electrical-to-mechanical energy transfer, while the electrical impedance measurement validated the back-reflection of the mechanical response into the electrical domain. 

## 4. Experiment Results and Discussions 

### 4.1. Optical Inspection

**Figure 7:**
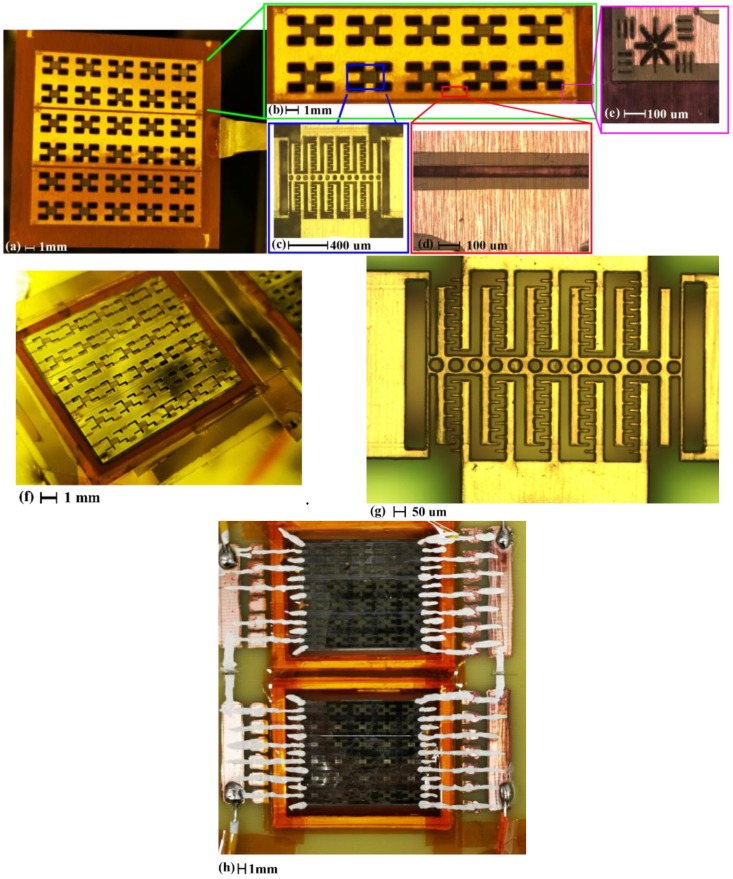
Optical views of the proof-of-concept transducers. (**a**) After releasing through copper etching; (**b**) Zoom-in view of 10 structures; (**c**) Zoom-in view for one structure after releasing before sputtering; (**d**) to (**e**) Overhangs formed by copper etching at different locations; (**d**) at the electrode separation gap; (**e**): at the left bottom corner; (**f**) 30 structures after sputtering; (**g**) Zoom-in view of one single transducer after sputtering; (**h**) 60 proof-of-concept structures after packaging for electrical-mechanical characterization.

Optical images of the fabricated proof-of-concept structures are shown in Figure 7.

For the released structures in Figure 7a,b, no polyimide layers have remained underneath. Figure 7c shows the representative status of the proof-of-concept structures. No significant deformation can be observed, indicating a good cross-linking strength of the SU-8 structures. Figure 7d,e indicate that overhangs have formed during the copper wet etching, simultaneously with the release of the structures. The depth of the overhang is only around 100 µm, much smaller than the computed value using the labeled etch rate for 5 h immersion (1.3 mm to 1.5 mm), indicating a self-limiting nature of the etching process. 

One possible explanation links the self-limiting nature with the pressure drop during the liquid propagation in microfluidic channels [30]. Overhangs are formed by wet etching of the copper covered by the SU-8 layers. During this process, the copper etchant propagates in a gap defined by the bottom surface of the SU-8 layer and the top surface of the polyimide layer, which is similar to the liquid propagation within microfluidic systems [30]. Since the contact angle of water is 90° on SU-8 [31] and 80° on polyimide [32], the hydrophobic behavior will act towards stopping the advance of the etchant [30], limiting the depth of the overhang.

As shown in Figure 7f, the surface of the proof-of-concept structures was reflective, without any cracks, after the sputtering of aluminum, indicating a good deposition quality. This conclusion can be further supported by the zoom-in view for a single structure in Figure 7g. As shown in Figure 7h, the packaging was done using Kapton tapes to fix the structures on a PCB board. The pads and the electrodes on the samples are electrically interconnected using silver paint. 

### 4.2. Mechanical Resonance under Electrical Actuation

**Figure 8:**
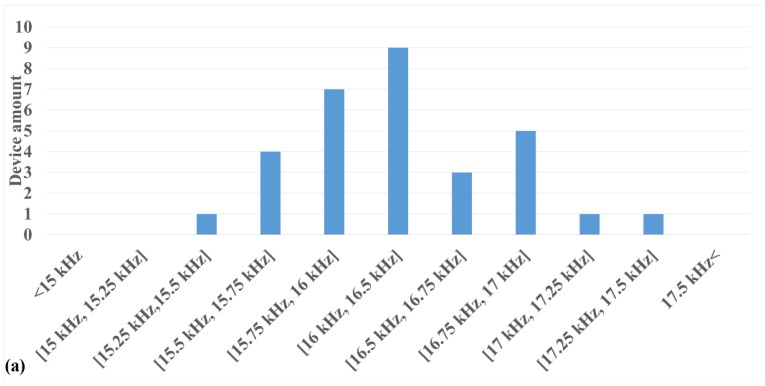

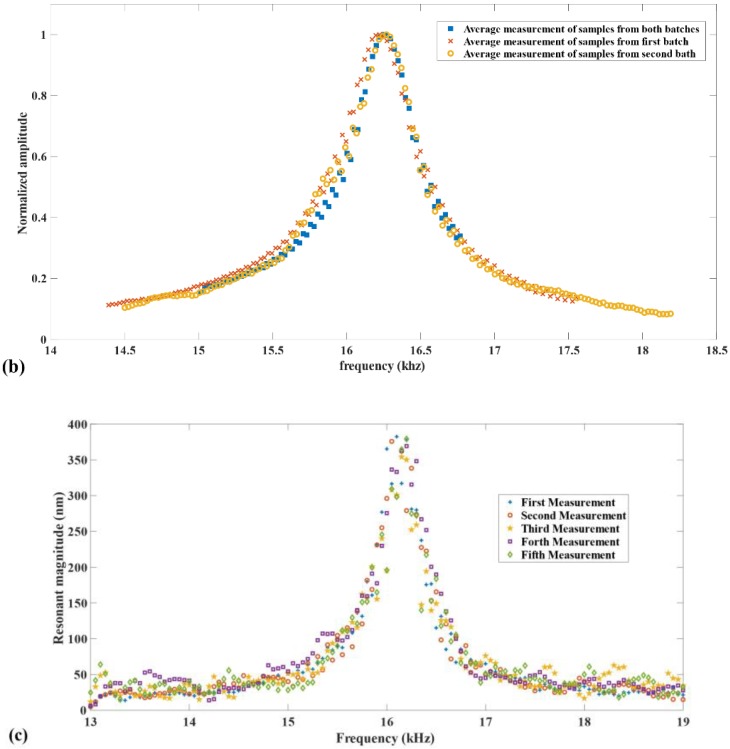
Visualization of the mechanical resonant measurement for the 30 selected proof-of-concept structures. (**a**) Distribution of resonant frequency of individual test structures. (**b**) Comparison of the average resonance profile between the two batches. (**c**) 5 separate measurement results for a single structure.

Table 3 provides the statistical information on the mechanical resonance measurements for the 30 selected test structures (15 from each batch), while the measurement result is illustrated in Figure 8. In addition, a video recording of the motion of a transducer during actuation is available as a separate supporting file. 

As shown in Table 3, the average error between the actual and simulated resonance frequencies is within 1%. The good matching between the measurement and simulation proves the high predictability of the fabrication technology. Meanwhile, the standard deviation of 3.24% indicates a dense distribution of the resonant frequency around the average value for each individual transducer, supporting a good reproducibility of the fabrication technology. 

Figure 8a shows the distribution of the measured resonant frequencies among the 30 measured test electrically actuated resonators, close to a normal distribution profile. In Figure 8b, the averaged mechanical resonance responses of the 15 test structures from each batch are compared. Besides a very minor variation in the resonant frequency, the averaged measurements of the two batches also have similar profiles. These results provide extra evidence for the good batch-level reproducibility of the fabrication technology. In Figure 8c, five repeated measurements were performed on the same structure, showing the stability and consistency of the measurement results. Overall, the presented predictability, reproducibility and controllability demonstrate the general robustness of the newly designed fabrication flow. 

### 4.3. Electrical Impedance Amplitude Measurement Results

**Figure 9:**
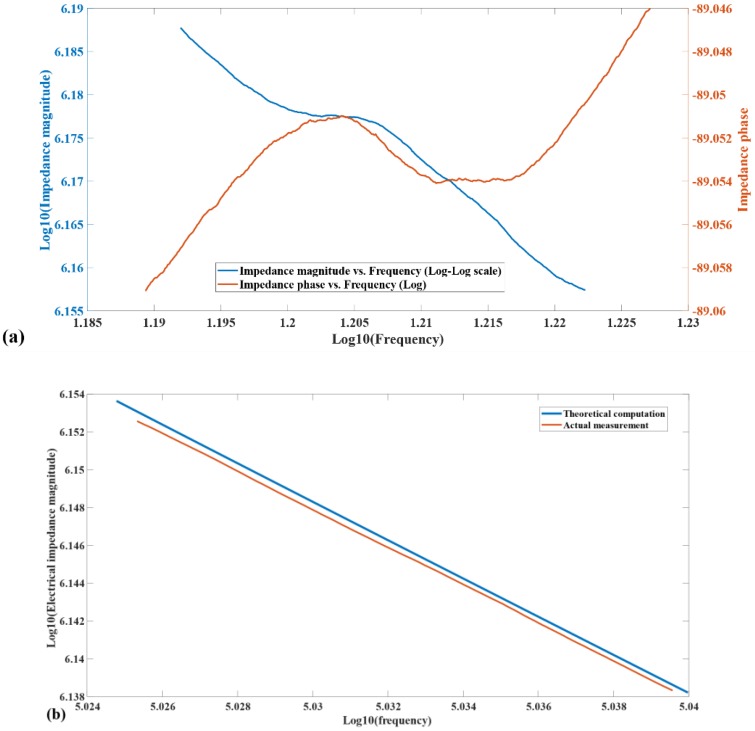
Electrical impedance characteristics of 60 in-plane test structures. (**a**) Impedance magnitude (log scale) vs. frequency (log scale) and Impedance phase vs. frequency (log scale) around the mechanical resonant frequency (around 16.25 kHz); (**b**) Impedance magnitude vs. frequency (log-log scale) away from the mechanical resonant frequency (16.86 kHz to 17.6 kHz).

The electrical impedance measurements of 60 fabricated test structures connected in parallel are shown in Figure 9. 

As shown in Figure 9a, around the mechanical resonant frequency, both the impedance magnitude-frequency and phase-frequency characteristics of the fabricated in-plane electrostatic test structures show a dominant capacitive behavior, with a mechanical resonance back-reflected into the electrical impedance value, influenced by parasitic effects and losses related to the electrical interconnects. The appearance of the back-reflection of the mechanical resonance verifies the existence of the mechanical-to-electrical coupling interface in the test structures. 

When the frequency of the electrical actuation signal is away from the mechanical frequency, such as the frequency band in Figure 9b, the reflection of the mechanical behavior back into the electrical domain becomes negligible. Consequently, the following approximation, for small signal actuation levels, of the impedance magnitude, becomes reasonable:

(2) $$\left\{ \begin{array}{l} \left|Z(\omega j)\right|=\frac{1}{C_{0}\omega }\\ \log_{10}(\left|Z(\omega j)\right|)=-\log_{10}(C_{0})-\log_{10}(\omega ) \end{array}\right.$$

In Equation (2), *Z(ωj)* is the electrical impedance; *C*_0_ is the estimated initial capacitance of 60 in-plane SU-8 electrostatic test structures; *ω* is the frequency of the actuation signal. On the log–log plot of the impedance magnitude vs. frequency curve, the vertical shift between the theoretical computation using Equation (2) and the actual measurement directly reflect the difference between the initial capacitance theoretically assumed and the one achieved through the actual fabrication process. In Figure 9b, the vertical shift between the two log–log scale curves corresponds to a capacitance difference of less than 1%, experimentally validating the effectiveness of the tilted magnetron sputtering in coating the vertical walls in a relatively comprehensive way. 

## 5. Conclusions

This paper introduces a simple and robust four-step fabrication flow for SU-8 in-plane capacitive MEMS structures. In comparison with existing fabrication flows for similar structures, the new technology reduces the processing complexity by at least two steps. Using copper-polyimide composites as the substrate, the fabrication flow intends to enhance the application potential of SU-8 MEMS structures in disposable/low-cost electronics, wearable MEMS, etc. The designed process flow enables the direct, laser-based micromachining of the polyimide substrates in a more practical way, resulting in extra process safety and simplicity. Within a single copper wet etching step, the overhang structures to avoid electrical short circuits are created, while the in-plane movable MEMS structures are released and suspended within an open window on the substrate, with an improved mechanical performance (e.g. reduced air-structure damping). 

To experimentally validate the feasibility of the newly developed fabrication flow, after the selection of materials, experimental calibrations were carried out to determine critical processing and design rules. Then, in-plane SU-8 test MEMS structures were designed, fabricated and characterized. Through the characterization of the fabricated test structures, the fabrication flow exhibited good process controllability, acceptable reproducibility (performance variation < 4%), high predictability (relative error < 1%) and a general robustness. In addition, the experimental acquisition of the corresponding measurement data verified the existence of the bi-directional electro-mechanical interface for actuation and sensing even in the simple test structures fabricated, validating the basic usability of the newly developed fabrication flow. With process qualities validated through the experimental case study, the newly developed, four-step fabrication flow offers promising perspectives for the development of polymeric inertial MEMS sensors for many cost-critical applications. 

## Figures and Tables

**Table 1:** Processing parameters of the 75 µm SU-8 2050 for experimental validation.

Soft Baking		Post-Exposure Baking		Developing
65 °C	95 °C	65 °C	95 °C	6 min (Immersion in SU-8 developer)
3 min	5 min	3 min	5 min	

**Table 2:** Structural parameters of the in-plane SU-8 MEMS test structures.

Actuation/Sensing Principle:				Area Variation		
Interdigitated Plates	Length (um)	Overlapping Length (um)	Width (um)	Thickness (um)	Gap (um)	Amount
	50	25	10	75	25	100
Ridge	Length (um)		Width (um)	Thickness (um)		Amount
	350		50	75		12
Elastic beam	Length (um)		Width (um)	Thickness (um)		Amount
	375		20	75		4
Rigid mass	Length (um)		Width (um)	Thickness (um)	Releasing hole radius (um)	Releasing hole amount
	1300		100	75	30	13
Estimated coupling capacitance of an individual test structure (fF)			110.63			
**Material properties of SU-8 2050 used for simulation**						
Young’s Modulus (GPa)			Poisson’s ratio		Density (kg/m^3^)	
4.95 [27]			0.33 [28]		1200 [29]	

**Table 3:** Statistical information of the measurement result for mechanical resonance.

Average Frequency Measured (kHz)	Standard Deviation	Relative Error to Simulation	Average Quality Factor
16.2590	3.2%	−0.92%	60.82
